# Women's knowledge about cervical cancer risk factors, screening, and reasons for non-participation in cervical cancer screening programme in Estonia

**DOI:** 10.1186/1472-6874-11-43

**Published:** 2011-09-28

**Authors:** Alice Kivistik, Katrin Lang, Paolo Baili, Ahti Anttila, Piret Veerus

**Affiliations:** 1 Department of Epidemiology and Biostatistics, National Institute for Health Development, Hiiu 42, 11619 Tallinn, Estonia; 2 Department of Public Health, University of Tartu, Ravila 19, 5041 Tartu, Estonia; 3 Descriptive studies and health planning unit, Fondazione IRCCS "Istituto Nazionale dei Tumori", Via Venezian 1, 20133 Milan, Italy; 4 Finnish Cancer Registry, Institute for Statistical and Epidemiological Cancer Research, Pieni Roobentinkatu 9, FI-00130 Helsinki, Finland

**Keywords:** cervical cancer screening, Estonia, reasons for non-participation

## Abstract

**Background:**

The attendance rate in Estonian cervical cancer screening programme is too low therefore the programme is hardly effective. A cross-sectional population based survey was performed to identify awareness of cervical cancer risk factors, reasons why women do not want to participate in cervical screening programme and wishes for better organisation of the programme.

**Method:**

An anonymous questionnaire with a covering letter and a prepaid envelope was sent together with the screening invitation to 2942 randomly selected women. Results are based on the analysis of 1054 (36%) returned questionnaires.

**Results:**

Main reasons for non-participation in the national screening programme were a recent visit to a gynaecologist (42.3%), fear to give a Pap-smear (14.3%), long appointment queues (12.9%) and unsuitable reception hours (11.8%). Fear to give a Pap-smear was higher among women aged 30 and 35 than 50 and 55 (RR 1.46; 95% CI: 0.82-2.59) and women with one or no deliveries (RR 1.56, 95% CI: 0.94-2.58). In general, awareness of cervical cancer risk factors is poor and it does not depend on socio-demographic factors. Awareness of screening was higher among Estonians than Russians (RR 1.64, 95% CI: 1.46-1.86). Most women prefer to receive information about screening from personally mailed invitation letters (74.8%).

**Conclusions:**

Women need more information about cervical cancer risk factors and the screening programme. They prefer personally addressed information sharing. Minority groups should be addressed in their own language. A better collaboration with service providers and discouraging smears outside the programme are also required.

## Background

Estonia is one of the countries in Eastern Europe with the highest incidence and mortality rates for cervical cancer. In the year 2008, the estimated world age standardised incidence rate of cervical cancer was 19.1 per 100, 000 women-years in Estonia with 151 new cervical cancer cases being detected [1,2]. The 5-year age-standardised relative survival among cervical cancer patients diagnosed from 1990 to 1994 is 63% in Europe, and 53% for Estonia [3]. In last 25 years, the cervical cancer incidence and mortality rate in Estonia have not decreased [4]. Every year about 143-190 new cervical cancer cases are detected and about 70 women die from this disease (Estonian Cancer Registry, unpublished data). Among all malignant tumours, cervical cancer is the one which can be most effectively controlled by organised screening programmes [5]. Previous studies have concluded that the lack of cervical smear history or poorly validated screening services [6] is the major reason why the disease still occurs [7].

Organised nationwide screening for cervical cancer was started in Estonia in 2006, before that, pilot studies took place from 2003 to 2005 [8]. Women in the age group of 30 to 59 with health insurance get invitations to screening with a 5 year-interval after a negative test. Women diagnosed with cervical cancer, women without health insurance and women for whom the Pap-test has been reimbursed in the last 12 months are excluded from the list of invitees. In 2010 there were 8.1% of women at age 30 to 59 without health insurance- most of them long term unemployed. Trained midwives take Pap-tests of the programme in 19 clinics all over Estonia and cytological analyses are made in 7 laboratories. In order to get the results, women have to contact the clinic on a certain date and time [8]. The major problem is low participation in the screening programme. In 2006, the attendance rate was 20.7% [9], and it slowly increased to 36% in 2009. Pap-smears taken within organised screening programme constitute less than 10% of all tests, most of the Pap-tests are taken by gynaecologists during a regular health control. But an organised screening programme can reduce incidence and mortality by 80% [10] as shown in other European countries. Another major shortcoming of the system is the lack of an electronic screening registry, which would make it easier to collect test results and monitor women with an abnormal smear.

The present article shows results of a survey addressed to women who were invited to a cervical screening programme in order to obtain information on their awareness about cervical cancer risk factors and screening, wishes for better organisation of the screening programme, and reasons for non-attendance in the organised screening programme.

## Methods

### Sample and data collection

In 2010, the Estonian Health Insurance Fund mailed 37, 275 personal invitations to women aged 30 to 55 to participate in a cervical cancer screening programme. Invitations, based on the data from the Estonian Population Registry, were sent to women born in 1955, 1960, 1965, 1970, 1975, and 1985. Of those women, a random sample of 3, 047 was selected to receive an anonymous questionnaire with the invitation. For various reasons, the questionnaires were not sent out to all women: 39 women were excluded because they had lost health insurance, 3 women had died and 63 women had an incomplete address in the Population Registry. For randomisation we used women's study identification numbers in Excel randomization system. By comparison of socio-demographic characteristics (age, ethnicity and place of residence) between the sample and Statistics Estonia population data we considered this sample representative.

The final sample comprised 2, 942 women who received a questionnaire and a prepaid envelope with a screening invitation. All women got questionnaires both in the Estonian and in the Russian language. Questionnaires and invitations were posted between 29 March and 2 April 2010. In December 2010, 1, 600 questionnaires with reminders were mailed to women who had not attended the screening programme after the first mailing. To check who had already participated in the screening programme, personal identification codes of the women included in the sample were linked with the Estonian Health Insurance Fund database. Women who had already fulfilled the questionnaire first time and received it again, were asked to ignore it.

The questionnaire included 21 questions and was divided into four sections (additional file 1): awareness about cervical cancer screening and risk factors for cervical cancer; reasons for non-participation in the national cervical cancer screening programme; women's preferences for the organisation of the cervical cancer screening programme; socio-demographic background data of the respondents [11]. The study design was approved by the Tallinn Medical Ethics Committee.

### Data analysis

For statistical analysis software package STATA 10 was used.

Basic descriptive statistics and frequencies were analysed for all variables.

Relative risk ratios were estimated by log-binomial regression to assess the association between knowledge of the screening programme, knowledge of cervical cancer risk factors, and reasons for non-participation (dependent variables) and socio-demographic characteristics (independent variables) that include mother tongue/ethnicity, place of residence, age, and number of children.

The "ethnicity" variable was categorised into 2 groups: Estonians and non-Estonians (the latter consisted of mainly Russian speaking population). Russian-speaking women are those who returned the questionnaire in Russian. Place of residence was also coded as a binary variable: urban areas (big cities and small towns) and rural areas (villages and settings). For certain analyses, age-groups of 30 and 35, 40 and 45, 50 and 55 were combined, respectively.

## Results

A total of 2, 942 questionnaires were mailed and after a reminder, 1, 054 women (36%) returned the completed questionnaire. The mean age of the respondents was 43 years; 74.3% were married or cohabiting, 10.5% were single and 14.5% were widowed or divorced; 69.1% were Estonian; 76.9% were working, 10.4% were housewives, 11.9% were retired or unemployed; and 69.0% lived in urban areas (Table 1). From background characteristics, it was possible to compare respondents and non-respondents only by age, and there was no difference between these two groups in that respect (data not shown).

**Table 1:** Socio-demographic characteristics of women participating in the study

Variable	N = 1054	Proportion (%)
**Age group, yrs**		

30	130	12.5

35	155	14.9

40	168	16.2

45	173	16.7

50	194	18.7

55	217	20.9

*Missing*	17	1.6

**Ethnicity/mother tonge**		

Estonian	728	69.1

Non-Estonian*	308	29.2

*Missing*	18	1.7

**Marital status**		

Married or cohabiting	783	74.3

Single	111	10.5

Divorced/widowed	158	14.5

*Missing*	2	0.2

**Occupational situation**		

Working	811	76.9

House wife	110	10.4

Student	6	0.6

Retired	38	3.6

Unemployed	87	8.3

*Missing*	2	0.2

**Place of residence**		

Urban	728	69.0

Rural	322	30.5

Missing	4	0.4

**Number of children**		

0	121	11.6

1	254	24.3

2	459	43.8

3	157	15.0

≥4	56	5.4

*Missing*	7	0.7

* mainly Russian speaking population

72.3% of the women answered that they were aware of the cervical cancer screening programme. In the adjusted model screening awareness depended on ethnicity - Estonian-speaking women were better aware of the programme than the others (RR 1.64; 95% CI: 1.46-1.86). Women with more than two deliveries had a slightly better knowledge about screening (RR 1.09; 95% CI: 1.00-1.18) (Table 2).

**Table 2:** Women's awareness of cervical cancer screening programme in Estonia

Socio-demographic characteristics	N (yes/no)	RR(CI 95%)	Adjusted* RR (CI 95%)
**Age group, yrs**			

30 and 35	198/87	1	1

40 and 45	257/83	1.08 (0.99-1.20)	1.04 (0.95-1.14)

50 and 55	291/12	1.02 (0.92-1.13)	1.04(0.96-1.14)

**Ethnicity/mother tongue**			

Non-Estonian	152/156	1	1

Estonian	593/134	1.65 (1.47-1.86)	1.64 (1.46-1.86)

**Place of residence**			

Urban	498/229	1	1

Rural	259/63	1.17 (1.09-1.26)	1.02 (0.96-1.10)

**Number of children**			

0-1	248/126	1	1

≥2	505/167	1.13 (1.04-1.23)	1.09 (1.00-1.18)

* Adjusted for all the dependent variables: age, ethnicity/mother tongue, place of residence, number of children.

**Figure 1:**
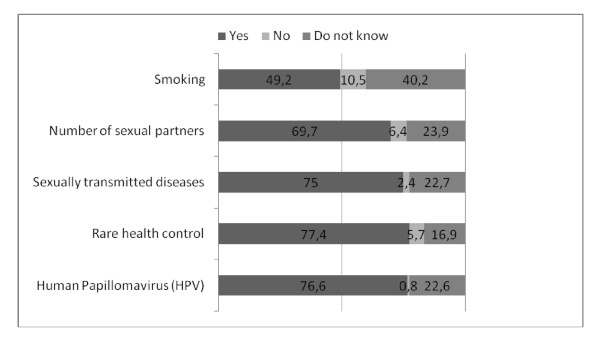
**Estonian women's awareness of cervical cancer risk factors (N = 1054), 2010**.

In the questionnaire all the cervical cancer risk factors were given without stating this and women were asked whether they think that these are risk factors or not. Women did not have a good overview of the impact of smoking as a cause of cervical cancer - only 49.2% of the women marked this as a risk factor. HPV was better known as a risk factor - 76.6% of the women marked it (Figure 1). Awareness of the risk factors for cervical cancer did not depend on socio-demographic factors (data not shown).

75.7% of the women responded that they were planning to participate in a cervical cancer screening programme. The wish to participate in the screening programme tended to be higher in the older age groups (Table 3).

**Table 3:** Wish to participate in cervical cancer screening programme among Estonian women, 2010

Variable	N (yes/no)	RR (95%CI)	Adjusted*RR(95%CI)
**Age group, yrs**			

30 and 35	206/76	1	1

40 and 45	276/65	1.12 (1.07-2.28)	1.13 (1.03-1.23)

50 and 55	302/10	1.06 (0.75-1.50)	1.03 (0.93-1.13)

**Ethnicity/mother tongue**			

Estonian	547/175	1	1

Non-Estonian	233/74	1.00 (0.93-1.08)	1.02 (0.94-1.10)

**Place of residence**			

Urban	550/174	1	1

Rural	241/78	0.99 (0.92-1.07)	1.02 (0.94-1.11)

**Number of children**			

0-1	281/93	1	1

≥2	508/158	1.02 (0.94-1.09)	0.99 (0.92-1.06)

* Adjusted for all the dependent variables: age, ethnicity/mother tongue, place of residence, number of children.

Among the respondents, 254 (24.3%) women answered that they were not planning to participate in the cervical cancer screening programme. Next we asked more general questions regarding all possible reasons for women's non-attendance and not only for non-attendance in this particular year. 41.9% (442) of the women pointed out different reasons why they could not participate in screening. The most common reason for this was a recent health control at a gynaecologist (42.3%). Other reasons were fear to give a Pap-smear (14.3%), long waiting list for appointment (12.9), clinic is far away (12.7) and unsuitable reception times (11.8%). Approximately 58.1% of the women did not answer this question, (Table 4).

**Table 4:** Reasons for non-attendance in the cervical cancer screening programme (N = 442)

Reasons	N	%
Recent visit to gynaecologist	187	42.3

Fear to give Pap-smear	63	14.3

Waiting list is too long	57	12.9

Clinic is far away	56	12.7

Appointment times not suitable	52	11.8

Do not have time	46	10.4

Uterus is removed	36	8.14

It is not necessary for me	9	2.0

Other reasons	52	11.8

A recent visit to a gynaecologist was more likely the reason for non-participation in the screening programme among Estonian than non-Estonian women (RR 2.00, CI 95%: 1.39-2.88) and married/cohabiting women (RR 1.67, CI 95%: 1.17-2.38). The wish not to attend was not associated with place of residence or a recent visit to a gynaecologist.

Fear to give the Pap-smear was higher among 30- and 35-year-old women than in 50- and 55-year-old women (RR 1.50, 95% CI: 0.81-2.78). Estonian women found it more likely than others that appointment times are not suitable (RR 5.76; 95% CI: 2.08-16.04), the same was found for women from urban areas (RR 1.98; 95% CI: 1.05-3.76). Women from urban areas also found appointment queues too long (RR 1.64, 95% CI: 0.86-3.13).

Most women prefer the current system to register by phone for the reception to give the Pap-smear (83.2%). 13.0% prefer to register by e-mail and nearly as many women (10.4%) expressed their wish to register by webpage. Women prefer to give a Pap-smear at a women's clinic rather than at a general practitioner (92.1%). To receive Pap-smear results, women equally prefer phone (33.9%) and e-mail (34.3%). The most uncomfortable method to get results was stated to be by SMS (3.9%).

Most of the women (74.8%) expect to receive information about cervical cancer screening programme from personally sent invitations with information leaflets. 18% prefer thematic articles and advertisements in women's magazines and 19% would like to get information from family practitioners. Only 9.8% prefer advertisements on television, and 5.8% prefer to receive no information. 89% of the women would feel happy to receive reminders from their general practitioner to participate in the screening.

## Discussion

This study had three principal aims. First, to estimate which socio-economic characteristics are associated with limited knowledge about cervical cancer screening programme and risk factors for cervical cancer. Secondly, to investigate reasons why so many Estonian women do not participate in the cervical cancer screening programme. And finally, to study women's preferences for better organisation of the screening programme.

An important outcome of our study was that approximately a quarter of the respondents were not at all or were only partially aware of cervical cancer screening. According to other studies, the awareness was lower among minority groups, and also in our study the Russian-speaking respondents were less aware of the screening, although invitations and information sheets are bilingual.

The study results revealed that there is a strong need to improve women's knowledge about cervical cancer risk factors. Women do not know enough about the main cervical cancer risk factors, particularly low is knowledge about the impact of smoking. Still, knowledge about cervical cancer risk factors is higher than many other studies published to date [12].

Our study results show that most respondents were satisfied with the present organisation of the screening programme. Most respondents prefer to have the Pap-smear taken by a gynaecologist or a midwife rather than by general practitioners, the way it has been arranged in Estonia. Additionally, over 90% of them are happy if general practitioners or nurses remind them to participate in the screening programme. Women prefer to register for screening by phone, but would like to learn about the results equally well by either phone or e-mail thus, to use e-mail for delivering results should be considered. Women prefer personally addressed information sharing, i.e. personal invitations with information leaflets. Although some studies have shown that giving the appointment time and place readily in the invitation letter would increase attendance [13], there is no need to do it Estonia because women are satisfied with the current system. Still, there might be a need to do some additional organisational changes regarding the screening programme, for example, routinely sending a reminder to all non-attenders, which has not been done in Estonia regularly although a systematic review shows its effectiveness [14], and also self-sampling could be tried [15].

According to our results, the main reason why women do not attend the screening programme is a recent visit to a gynaecologist (42.3%). This shows a clear need to improve collaboration between service providers and discourage Pap-smears outside the organised screening programme. Also, another very important reason for non-attendance needs to be noticed - women have fear to give Pap-smears. Previous studies have also shown that the fear to give Pap-smear is one barrier for screening [16]. According to this result, we are not able to say exactly what this fear means to women - whether it is fear for cancer or fear for Pap-smear procedure, but there is a clear need to better inform women about the screening procedure and cervical cancer. Some earlier studies have shown that women who do not attend screening have higher fear and anxiety for cancer [17].

Women from urban areas pointed out unsuitable appointment times and too long appointment queues, which could mean that health service availability is not good enough in urban areas. More resources need to be addressed to develop invitational and sample-taking activities and units. Yet there is no available data of how many women actually attended a screening after receiving a remainder.

Our study had some limitations. One of the most important shortcomings of this survey was the low response rate, probably concerning especially women who are not planning to participate in screening. This is a common problem in studies among non-attenders or in a population with a very low attendance rate in screening. In the early stage of planning the study, we had to consider the fact that women who are most likely to respond to the questionnaire are the ones who wish to participate in the screening or have already been to a gynaecologist - because they have more interest in the subject. This tendency has been noted in several studies before [18]. Another problem was that women often did not respond to the question about reasons of non-attendance.

Also, the education level was not asked from respondents, but earlier studies have shown important association between health behaviour and educational level [19].

## Conclusion

There is a clear need for better information sharing about cervical cancer risk factors and screening in the total screening population. Russian-speaking women, older women and women with a smaller number or no deliveries need special attention. To increase effectiveness of the program and reduce cervical cancer burden, there is a need to decrease Pap-smears taken outside screening program. Special attention should be paid to availability of screening services in urban areas. Instead of big information campaigns, women in Estonia prefer individualised and delicate information sharing; this should be taken into account while tailoring the campaigns and invitations.

## Competing interests

The authors declare that they have no competing interests.

## Authors' contributions

PV, KL, PB, AA designed the study. PV conducted the data collection and analyses, and completed the initial draft of paper together with AK. All authors read and approved the final manuscript.

## Pre-publication history

The pre-publication history for this paper can be accessed here:

http://www.biomedcentral.com/1472-6874/11/43/prepub

## Supplementary Material

Additonal file 1 **Questionnare used for survey Women's knowledge about cervical cancer risk factors, screening, and reasons for non-participation in cervical cancer screening programme in Estonia**. Click here for file
