# Supplementary material for: Women's knowledge about cervical cancer risk factors, screening, and reasons for non-participation in cervical cancer screening programme in Estonia
Source: BMC Womens Health. 2011 Sep 28;11:43. doi: 10.1186/1472-6874-11-43 (PMC3192747; doi:10.1186/1472-6874-11-43)
Supplement: Additonal file 1 — Questionnare used for survey Women's knowledge about cervical cancer risk factors, screening, and reasons for non-participation in cervical cancer screening programme in Estonia. [file 1472-6874-11-43-S1.DOC]

QUESTIONNAIRE

1. Have you heard about the cervical cancer screening program?

1 yes

2 no

1. Do you fully understand the enclosed invitation and leaflet describing the screening programme?

1 yes

2 no

1. Do you plan to participate in the cervical cancer screening programme?

1 yes

2 no

1. Listed below are some possible reasons for non-attendance, please encircle as many as needed

1 I have just had a regular check-up at my gynaecologist

2 I do not have a time for it

3 the reception hours are not suitable

4 the clinics is too far from my living-place

5 the waiting-time is too long

6 I am afraid to give a test

7 my uterus has been removed

8 I do not think it is necessary

1. How would you like to register for the screening?

1 by phone

2 by e-mail

3 via web

1. Where would you like to have the Pap-smears taken?

1 at the women´s clinic

2 at the family doctor´s office

1. How would you like to be informed about your test result?

1 by phone

2 by mail

3 by e-mail

4 from a midwife or a doctor

1. When did you last visit your gynaecologist?

1 less than a year ago

2 less than five years ago

3 more than five year ago

4 don´t remember

1. If your family doctor would remind you about participation in the screening, how would you feel?

1 happy that he/she is concerned about my health

2 I don´t care

3 I wouldn´t like it

4 I don´t know

1. Where would you like to get information about the screening programme?

1 from TV

2 from women´s magazines

3 from family doctor/family nurse

4 together with a personal invitation sent by mail

5 other

6 I do not need more information

1. Which of the following factors are risk factors for cervical cancer screening?

|  | Yes | No | I don´t know |
| --- | --- | --- | --- |
| Smoking |  |  |  |
| Many sexual partners |  |  |  |
| HPV |  |  |  |
| No regular check-ups |  |  |  |
| STD |  |  |  |

1. Your age… years
2. Your nationality…
3. What is your marital status?

1 married

2 single

3 divorced

4 widowed

1. Are you currently in paid work?

1 yes

2 I am retired

3 I am unemployed

4 I study

5 other

1. Are you a daily smoker?

1 yes

2 no, I have never smoked

3 no, but I have been daily smoker earlier

1. How many times have you given birth?....
2. Have you ever had sexually transmitted diseases (gonorhhoea, chlamydiosis, trichomonosis)?

1 yes

2 no

3 don´t know

1. Have you ever used contraceptive pills?

1 yes

2 no

3 don´t know

1. How many sexual partners have you had in your lifetime?...
2. Your place of residence?

1 big town

2 small town

3 countryside
